# Biochemical Constituent of *Ginkgo biloba* (Seed) 80% Methanol Extract Inhibits Cholinesterase Enzymes in Javanese Medaka (*Oryzias javanicus*) Model

**DOI:** 10.1155/2020/8815313

**Published:** 2020-09-23

**Authors:** Ibrahim Hassan, Wan Norhamidah Wan Ibrahim, Ferdaus Mohamat Yusuf, Siti Aqlima Ahmad, Syahida Ahmad

**Affiliations:** ^1^Department of Biochemistry, Faculty of Biotechnology and Biomolecular Sciences, Universiti Putra Malaysia, UPM Serdang, Seri Kembangan 43400, Selangor, Malaysia; ^2^Department of Biology, Faculty of Science, Universiti Putra Malaysia, UPM Serdang, Seri Kembangan 43400, Selangor, Malaysia; ^3^Department of Environmental Science, Faculty of Environmental Study, Universiti Putra Malaysia, UPM Serdang, Seri Kembangan 43400, Selangor, Malaysia

## Abstract

**Background:**

Pathophysiological changes leading to the death of nerve cells present in the brain and spinal cord are referred to as neurodegenerative diseases. Presently, treatment of these diseases is not effective and encounters many challenges due to the cost of drug and side effects. Thus, the search for the alternative agents to replace synthetic drugs is in high demand. Therefore, the aim of this study is to evaluate the anticholinesterase properties of *Ginkgo biloba* seed.

**Methods:**

The seed was extracted with 80% methanol. Toxicity studies and evaluation of anticholinesterase activities were carried out in adult Javanese medaka (*Oryzias javanicus*). Phytochemical study to identify the bioactive lead constituents of the crude extract was also carried out using high performance liquid chromatography (HPLC).

**Results:**

The result shows activities with high significant differences at *P* < 0.001 between the treated and nontreated groups. A bioactive compound (vitaxin) was identified with the aid of HPLC method.

**Conclusion:**

The presence of bioactive compound vitaxin is among the major secondary metabolites that contribute to increasing activities of this plant extract. High anticholinesterase activities and low toxicity effect of this plant show its benefit to be used as natural medicine or supplements.

## 1. Introduction

*Ginkgo biloba* (Gb) or maidenhair tree is commonly found in China and used for several medicinal purposes since time immemorial. It is one of the most used ancient seeds referred to as “living fossil,” tree that can survive for more than 1000 years and can reach a height of up to 40 m [1]. Leaf extract from this tree is traditionally used in Asia for centuries to treat circulatory disorders, asthma, tinnitus, vertigo, and cognitive problems [2]. Several reports show that extracts from different parts of this plant are used as supplements or brain stimulant which has been standardized worldwide as safe and most recommended medicinal plant. Different parts of the tree have been commercialized and used in the treatment of several ailments in developed countries especially in the elderly and amongst people with dementia as nootropic agents due to its high activities [3]. Cholinesterase inhibitory effect of the extract from this tree has been documented. Hence, *Ginkgo biloba* and memantine are classified as among antidementic agents since 2000 years ago [4]. Phytochemical study of extracts from Gb tree revealed the presence of several bioactive compounds mainly terpenoids, flavonol glycosides, and proanthocyanidins. The most available bioactive compounds present in this plant are flavonol glycosides (quercetin and catechin) and terpenoids (ginkgolides and bilobalides), which are reported as unique bioactive components of this tree due to their high neurocurative effects. The pharmacological effects as well as medicinal properties of this plant are due to the presence of terpenoids, flavonoids, and proanthocyanidins as reported by several scientists [5]. Experimental animal studies also discovered the mechanisms of action that have been postulated to reaffirm its pharmacological properties, ability to inhibit platelet-activating factor and enhance nitric oxide elimination from the body system. Subsequently, its effect on increased peripheral and cerebral blood flow was reported and believed to be one of its major mechanisms of action [6]. Modulations of different neurotransmitter systems by extract from different parts of this tree have also been documented. It acts as strong inhibitor of monoamine oxidase A and synaptosomal uptake of DA, 5-HT, and norepinephrine [7]. However, extracts from different parts of this tree show high free radical scavenger activities, neuroprotective effects, and antiapoptotic properties. The plant has high potential to inhibit amyloid-*β* neurotoxicity, protect against hypoxic challenges, and neutralize oxidative stress [8]. Several previous studies of this plant have mainly focused on the efficacy of this tree extract in the management of dementia. Although, inconsistent and controversial results have been documented, this may be due to the different types and concentrations of the available bioactive compounds [9].

Cholinesterase or choline esterase belongs to the esterase family; these include set of enzymes that break acetylcholine to choline-based esters [10]. Thus, the mechanisms of action of these enzymes are either of the two: speed up the hydrolysis of cholinergic neurotransmitters (acetylcholine) or metabolize acetylcholine into choline and acetic acid. These biochemical reactions are very vital to allow a cholinergic neuron to return to its normal resting state after activation [11]. For example, acetylcholine at a motor enclave triggers muscle contraction, for the muscle to relax afterward cholinesterase has to act to neutralize acetylcholine rather than remaining locked in a tense state [12].

Javanese medaka fishes belong to the genus Oryzias, commonly found in fresh and salt water in Asia. The genus has about 20 species that are small in size (usually less than 4 cm) and highly transparent [13]. They are mostly present in shallow nonmoving or slow moving stream near the shore or bank, often found in rice paddy, ponds, irrigation channels, and small creeks. Almost all species of this genus are surface swimmers with relatively large eyes at the sides and slightly flattened head that are easily recognized by looking down the swimming fish from above [14]. Among this genus *O. latipes* species has already been confirmed as one of the best experimental fish for several studies. The fish has been used as a unique model for molecular studies, genomic sequence, informatics study, and transgenic study [15]. Another two species of genus are *O. javanicus* and *O. dancena* that are among the popularly used models in environmental sciences research. They are commonly found in Southeast Asia due to their wide distribution in the region. They can easily be acclimatized and kept in both fresh and salt water, although the former prefers fishes that can withstand hyperosmotic environment and the latter prefers fishes that can withstand hypoosmotic environment [16].

Despite the increased use of *Ginkgo biloba* leaf and seed as a supplements and or medicinal agent, there is paucity of information on its toxicity and cholinesterase effects on animal's cells and tissues. Hence, the present study aims to evaluate the effect of *Ginkgo biloba* 80% methanol seed extract on cholinesterase activities in the brain of Javanese medaka.

## 2. Materials and Method

### 2.1. Plants Collection and Identification

*Ginkgo biloba* seed was purchased from wholesalers at Chinese Traditional Pharmacy at Puchong, Selangor, Malaysia. The herb was identified by botanists at the Institute of Bioscience (IBS), University Putra Malaysia (UPM), and voucher number was allocated (SK2894/15). The seed was washed and air-dried at room temperature 26 ± 1°C for two weeks.

### 2.2. Plant Extraction

*Ginkgo biloba* seed was cleaned and allowed to dry for two weeks at room temperature (26 ± 1°C) before crushing to semipowdered form (40–60 mesh). A total of 200 g of leaf sample was soaked for 3 days in 1000 mL of 80% methanol in flat bottom flasks (Sigma-Aldrich, USA). The powdered seed methanol mixture was shaken daily for three days at 26°C to obtain high crude extract; this procedure was repeated three times to extract all bioactive components of the seed. The extract obtained was then filtered with Whatman filter paper (1.5 Sigma-Aldrich, USA) and then concentrated to semisolid form at 42°C with a rotary evaporator (IKA® RV 10, USA). The resultant crude extract obtained was then weighed and transferred into sample bottles and stored at 4°C until required for use [17].

Percentage yield of the crude extract was calculated as the weight of the filtrate divided by the total weight of the grounded seed in semipowder multiplied by 100: Yield (%) = [wt of extract (g)/wt of plant material (g)] × 100.

### 2.3. Plants Sample Dilution and Dose Preparation

Stock solution was prepared by dissolving 100 mg of *Ginkgo biloba* crude seed extract in 1 mL of 100% DMSO (100 mg/mL). The use of DMSO was to solubilize the crude extract, since the extract is not absolutely soluble in aqua solvent. Preparation of substocks solution in microliter (*μ*g/mL) was done by diluting the stock solution to the concentration of interest using distilled water. Working solution was prepared from substock solution using twofold serial dilution with distilled water at eight concentrations ranging from 7.81 to 1000 *μ*g/mL in a 96-well microplate (Sigma-Aldrich, USA) [18]. DMSO (vehicle) was maintained at 0.1% in all concentration of extract.

### 2.4. Subacute Toxicity Test of Arsenic on Adult Javanese Medaka (*Oryzias javanicus*)

Subacute toxicity test of arsenic was carried out on adult J. medaka (length of 1.9–2.7 cm, weight of 0.1–0.3 g, and age of 6 months) for 48 hours. Dechlorinized tap water was used in this study as reported by the OECD guideline for testing of chemicals. The fishes were grouped into 6 with five fishes of both sexes per group. The fishes were randomly selected and exposed to a particular dose of arsenic in a 3-liter capacity rectangular glass aquarium. Five different concentrations of arsenic (0.5–4 mM) with three replicates alongside the control group were used. Mortality was monitored continuously and the fishes were considered dead when operculum movement was no longer detected and the fishes could not respond by touched with a glass rod. The dead fishes were immediately removed from the tank. After 24 hours, the exposed experimental fishes were transferred to new tanks containing clean chlorine-free tap water. The fish were fed prior to but not during the experimental period. During the experiment, the behavior of the experimental fishes was monitored regularly by taking note of abnormal behavior manifested and/or death [19]. The experiments were conducted in compliance with the internationally accepted principles for laboratory animals use and care as well as ethical clearance by Institutional Animal Care and Use Committee (IACUC), Universiti Putra Malaysia (ref. no. UPM/IACUC/AUP-R005/2016).

### 2.5. Subchronic Toxicity Test of Arsenic on Adult Javanese Medaka (*Oryzias javanicus*)

Subchronic toxicity test of arsenic was conducted in adult J. medaka (length of 1.9–2.7 cm, weight of 0.1–0.3 g, and age of 6 months) for 7 days in chlorine-free tap water as reported by the OECD. Five fishes of both sexes were randomly chosen and exposed to prepared arsenic dose concentrations (0.05, 0.15, 0.75, 1.75, 2.75, and 3.75 mM/L) in a 3-liter capacity rectangular glass aquarium. Three replicates each along with control were used for the test. Mortality was monitored continuously and the fishes were considered dead when they fail to respond to touch with glass rod, and there was no evidence of respiration. Dead fishes were immediately removed from the tank. After 24 hours, exposed fishes were transferred to new tanks containing chlorine-free tap water. The fish were fed prior to and during the experimental period. During the experiment, the fish were closely monitored [19]. The experiments were conducted in compliance with the internationally accepted principles for laboratory animals use and care as well as ethical clearance by Institutional Animal Care and Use Committee (IACUC), Universiti Putra Malaysia (ref. no. UPM/IACUC/AUP-R005/2016).

### 2.6. Subacute Toxicity Test of Plant Extract on Adult Javanese Medaka (*Oryzias javanicus*)

Subacute toxicity test of *Ginkgo biloba* seed crude extract was carried out on adult Javanese medaka (*Oryzias javanicus*) (length of 1.9–2.7 cm, weight of 0.1–0.3 g, and age of 6 months). The experiment was conducted in 2 days using chlorine-free tap water as reported by the OECD guideline for testing chemicals. Javanese medaka of both sexes was randomly selected and exposed to a particular dose of crude extract in a 3-liter capacity rectangular glass aquarium that contained 5 fish each. Five different concentrations (62.5–1000 mg/L) were used and each concentration has three replicates alongside the control group. Continuous monitoring of mortality was carried out and dead fishes were confirmed after the operculum movement was no longer detected and inability of the fishes to respond when touched with a glass rod. Dead fishes were immediately removed from the tank. The exposed fishes were then transferred to new tanks containing chlorine-free tap water after 24 hours of exposure. Fishes were feed before the experiment. The behaviors of fishes were monitored regularly during the experiment by taking note of abnormal behavior or sign of death [19]. The experiments were conducted in compliance with the internationally accepted principles for laboratory animals use and care as well as ethical clearance by Institutional Animal Care and Use Committee (IACUC), Universiti Putra Malaysia (ref. no. UPM/IACUC/AUP-R005/2016).

### 2.7. Chronic Toxicity Test of Plant Extract on Adult Javanese Medaka (*Oryzias javanicus*)

Chronic toxicity test of the crude extract was carried out on adult Javanese medaka (*Oryzias javanicus*) (length of 1.9-1.9–2.7 cm, weight of 0.1–0.3 g, and age of 6 months) for 2 weeks in chlorine-free tap water as reported by OCED. Random selection of J. medaka of both sexes was carried out and maintained at the stocking density of five fish per liter. The fishes were exposed to seven different concentrations of the crude extract (35, 45, 55, 65, 75, 85, and 95 mg/L). Three replicates each alongside the control groups were used for the test. The selection of the test concentration of crude extract was made based on the result obtained from acute toxicity tests of the crude extract on Javanese medaka. Survivals as well as behaviors of the fish were monitored closely during the experiment. The fishes were fed prior to and during the experimental period. During the experiment, the behaviors of the experimental fishes were monitored regularly [19]. The experiments were conducted in compliance with the internationally accepted principles for laboratory animals use and care as well as ethical clearance by Institutional Animal Care and Use Committee (IACUC), Universiti Putra Malaysia (ref. no. UPM/IACUC/AUP-R005/2016).

### 2.8. Screening of Cholinesterase Activities

Screening of cholinesterase activities of the crude extract on adult J. medaka (length of 1.9–2.7 cm, weight of 0.1–0.3 g, and age of 6 months) was carried out in chlorine-free tap water. Fishes were divided into four groups with 25 fishes per group and acclimatized for 2 weeks. The fishes were maintained in separate aerated 14-liter tanks and water was changed after every 24 hrs. The fishes were initially exposed to the safe dose of crude extract 50 mg/L for 24 hours and later exposed to 0.15 mM arsenic. These concentrations were chosen based on previous studies of acute, chronic toxicity test of crude extract as well as subacute and subchronic toxicity test of arsenic. The control groups were maintained in the same conditions but without arsenic and crude extract for 10 days. Three replicates were used for each concentration along with control groups [20].

At the end of the experiment, the fishes were cryoanesthetized with ice for 20 seconds. The fish head was gently decapitated and the brain was dissected and gently removed out without any damage. Brain sample was then washed with 50 mM Tris–HCl buffer. It was then weighed and homogenized in a scope bottle with tissue homogenizer (Polytron PT-6100, USA). Tris–HCl buffer (1% Triton X and 0.1% PMSF) (Sigma-Aldrich) was used as homogenizing solvent. Sample was centrifuged at 12,000 ×g for 20 minutes with (Grace High Speed Refrigerated Centrifuge, India). The supernatant was transferred into separate tube and used as enzyme source.

### 2.9. Total Protein Estimation

The protein content was determined on homogenized fish brain using Bradford method. Bovine serum albumin (BSA) 7.81–1000 *μ*g/mL was prepared and used as a standard. A 1 ml stock solution of 1000 *μ*g BSA/200 *μ*l Tris–HCl (10 mg/200 mL) pH 7.4 was diluted with Bradford reagent in 96-well microplate. Reading was taken at 590 nm using microplate reader (filter Berthold Technologies GmbH & Co. KG) and standard curve was plotted. The same was repeated for determination of total protein in the brain. Tris–HCl pH 7.4 was diluted with the brain sample at different concentrations from 0 to 1000 *μ*g/mL, 200 *μ*L of sterile phosphate saline (PBS); Bradford reagent was later added and then maintained for 30 minutes. Reading was taken at 590 nm using microplate reader (filter Berthold Technologies GmbH & Co. KG) [21].

After running the assay, the standard curve was used to extrapolate the protein concentration according to OD values.

### 2.10. Determination of Cholinesterase Activity of Plant Extract

A 50 mM Tris–HCl pH 7.4 was used as a buffer throughout the experiment. Cholinesterase (AChE) used in the assay was from the homogenized brain of experimental fishes. DTNB, ATC, BTC, and PTC were prepared in 50 mM Tris–HCl in 96-well plates I. A 210 *μ*L of Tris–HCl buffer (pH7.4), 20 *μ*L of 0.1 mM DTNB, and 10 *μ*L of AChE from the brain (54 mg/mL) were transferred into another 96-well plate II and incubated for 15 min at 28°C. Then, 10 *μ*L of ATC, BTC, or PTC (2.5 mM) was then added to the mixture in 96-well plate II and incubated for 10 minutes. Based on Ellman's method, anticholinesterase (ChE) activity was measured using a modified 96-well microplate assay. The hydrolysis of the substrate acetylthiocholine by cholinesterase enzyme results in the production of thiocholine. Thiocholine reacts with Ellman's reagent (DTNB) to produce 2-nitrobenzoate-5-mercaptothiocholine which was measured at 405 nm with microplate reader. Reading was taken at 405 nm using a microplate reader (Tecan Multimode Microplate, UK) at fluorescent excitation of 485 nm and 535 nm emission [22].

Cholinesterase (ChE) activities were calculated using the following formula:

(1) $$\begin{array}{c} \text{enzyme activity}\left(U\right)=\frac{\Delta \text{absorbance}/10\;\min}{\varepsilon }\times \left(\frac{\text{TV}}{\text{TS}}\right)µ\text{L of well}, \end{array}$$

where Δabsorbance = change in absorbance reading at 405 nm after 10 mn of incubation (final–initial) and *ε* = molar extinction coefficient = 0.0136 *μ*M^−1^ cm^−1^:

(2) $$\begin{array}{c} \text{TV}=\text{total volume}=250\;µ\text{L,}\\ \text{TS}=\text{total sample}=10\;µ\text{L.} \end{array}$$

### 2.11. Identification of Bioactive Compound

Plant bioactive compounds vitexin and isovitexin were used as standard in this experiment. The two standards were prepared at the concentrations of 70 to 4.4 *μ*g/mL and 97 to 6.1 *μ*g/mL. To identify the vitexin and isovitexin, crude extract was prepared at the concentration of 1 mg/mL.

### 2.12. High Performance Liquid Chromatography

HPLC system (Waters, USA), consisting of a 600 pump, an autoinjector, 2998 photodiode array detector 200 to 500 nm, was set up and used to determine the presence of bioactive compound (vitexin and isovitexin) in the crude extract. Separation was carried out using 250 × 4.6 mm ODS 3.3 mm column (Inertsil, Japan) thermostated at 40°C. A gradient method with methanol and deionized water was used for the separation. At 0 min, the mobile phase was set at 10% methanol in deionized water and increased to 90% methanol in deionized water for duration of 45 min. The 90% methanol in deionized water was maintained for further 15 min. The peaks were integrated at the wavelength of 337 nm [23].

### 2.13. Statistical Analysis

All experiments were repeated three times independently. Data were collected, processed, and interpreted as mean ± standard error of mean (mean ± SEM). Survival rate and anticholinesterase effect of the crude extract on *Oryzias javanicus* were analyzed using (GraphPad Software, USA). Statistical analysis using one-way ANOVA followed by Dunnett's post hoc test, where *P* < 0.05, was considered statistically significant as treated groups were compared with untreated groups (control) using GraphPad Prism version 5.0 software (GraphPad Software, USA).

## 3. Results

### 3.1. Result of Percentage Yield of the Crude Extract

Following extraction of grounded plant extract in 80% methanol and evaporated to semisolid form with a rotary evaporator at 42°C, the percentage yield obtained was 40.29 g.

### 3.2. Toxicity Test

#### 3.2.1. Result of Subacute Toxicity Test of Arsenic

**Figure 1:**
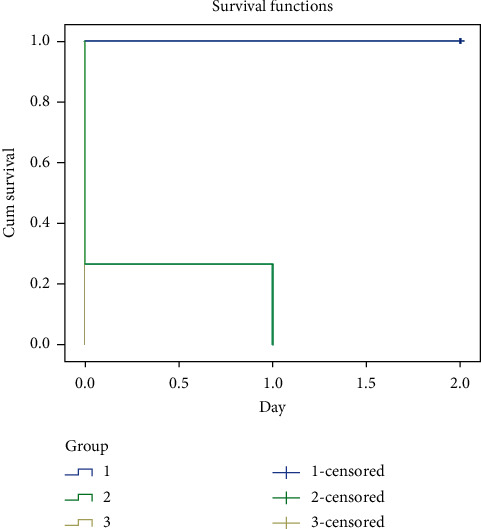
Subacute toxicity effect of arsenic on the survival rate of adult Javanese medaka treated with different concentrations of arsenic (0.5–4 mM). The percentage of survival rate is shown versus concentration of arsenic. ^*∗*^*P* < 0.001 represents significant different values of the control group and group exposed to arsenic. The values represent mean ± SD from three (*n* = 3) independent experiments.

The result of 2-day subacute toxicity test of arsenic on J. medaka shows high mortality at concentration above 0.5 mM. Only 20% of the fishes survived the second day of exposure at 0.5 mM. All fishes died at day 2 of the experiment at this concentration. All fishes at 1, 2, 3, and 4 mM concentrations died at day 1 of the experiment (Figure 1).

#### 3.2.2. Result of Subchronic Toxicity Test of Arsenic

**Figure 2:**
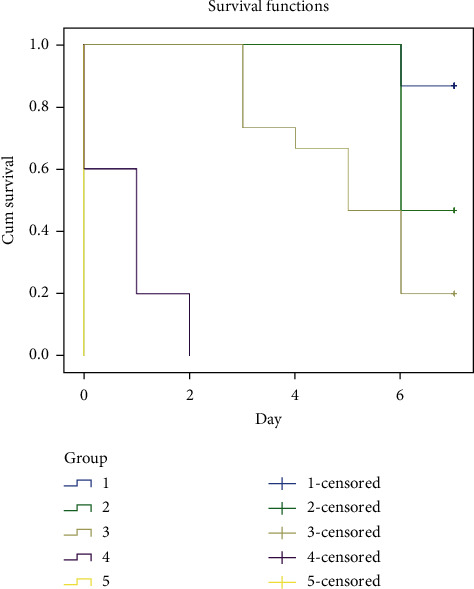
Subchronic toxicity effect of arsenic on the survival rate of adult Javanese medaka treated with different concentrations of arsenic (0.05–1.75 mM). The percentage of survival rate is shown versus concentration of arsenic. ^*∗*^*P* < 0.01 represents significant different values of the control group and group exposed to arsenic. The values represent mean ± SD from three (*n* = 3) independent experiments.

The result of 7-day subchronic toxicity test of arsenic on adult J. medaka shows high mortality at concentration above 0.15 mM. All the fishes at 0.75 mM concentration died at day 4 while those at 1.75 mM concentration died at day 1. At 0.15 mM concentration, 40% of the fishes survived till day 7 of the experimental study (Figure 2).

#### 3.2.3. Result of Acute Toxicity Test of Crude Extract

**Figure 3:**
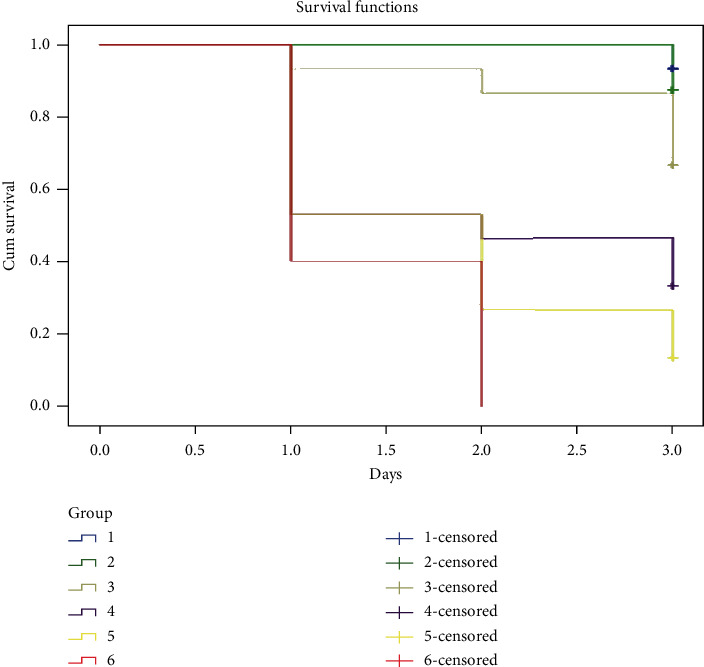
Acute toxicity test of crude extract on the survival rate of adult Javanese medaka treated with different concentrations of the crude extract (62.5–1000 mg/L). The percentage of survival rate is shown versus concentration of crude extract. ^*∗*^*P* < 0.01 represented significant different values of the control group and group exposed to crude extract. The values represent mean ± SD from three (*n* = 3) independent experiments.

The result of 2-day acute toxicity test of crude on J. medaka shows high mortality at concentration above 250 mg/L. At day 2, only 33% of the fishes survived at 250 mg/L, 7% survived at 500 mg/L, and all fishes at 1000 mg/L died (Figure 3).

#### 3.2.4. Result of Chronic Toxicity Test of Crude Extract

**Figure 4:**
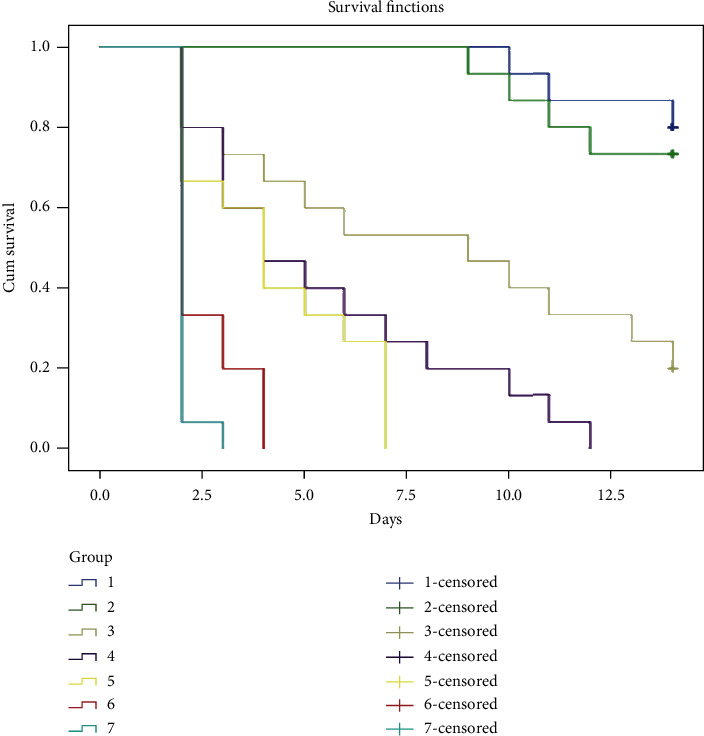
Chronic toxicity test of crude extract on the survival rate of adult Javanese medaka treated with different concentrations of the crude extract (100–400 mg/L). The percentage of survival rate is shown versus concentration of crude extract. ^*∗*^*P* < 0.01 represents significant different values of the control group and group exposed to crude extract. The values represent mean ± SD from three (*n* = 3) independent experiments.

The result of 14-day chronic toxicity test of crude extract on adult J. medaka shows high mortality at concentration above 150 mg/L. All the fishes at 250 mg/L concentration died at day 12, those at 300 mg/L died at day 7, those at 350 mg/L died at day 4, and those at 400 mg/L died at day 3 of the experimental study (Figure 4).

#### 3.2.5. Result of Total Protein

**Figure 5:**
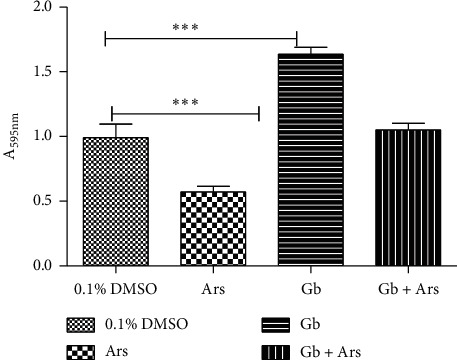
Total protein assay of adult Javanese medaka treated with arsenic, crude extract, and arsenic and crude extract. The percentage of total protein is shown versus concentration of the tested sample (arsenic, crude extract, and crude extract and arsenic). ^*∗*^*P* < 0.001 represents significant different values of the control group and group exposed to arsenic only, crude extract and arsenic, and crude extract only. The values represent mean ± SD from three (*n* = 3) independent experiments.

Result of total protein analysis shows a significant difference at *P* < 0.001 between the fishes that were maintained at tap water with 0.1% DMSO and the group that were exposed to arsenic. Significant difference at *P* < 0.001 was also observed between the fishes that were maintained in tap water and the fishes that were treated with only crude extract (Figure 5).

#### 3.2.6. Result of Acetylcholinesterase Inhibition

**Figure 6:**
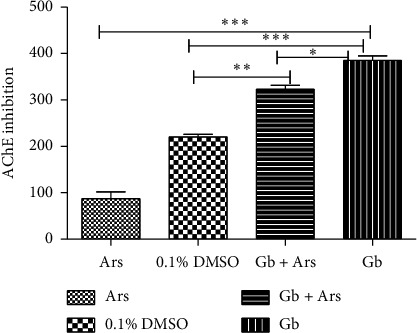
Acetylcholinesterase inhibition assay of adult Javanese medaka treated with arsenic, crude extract, and arsenic and crude extract. The percentage of acetylcholinesterase inhibition is shown versus concentration of the tested sample (arsenic, crude extract, and crude extract and arsenic). ^*∗*^*P* < 0.001, *P* < 0.01, and *P* < 0.05 represents significant different values of the control group and group exposed to arsenic only, crude extract and arsenic, and crude extract only. The values represent mean ± SD from three (*n* = 3) independent experiments.

Result of cholinesterase inhibition shows a significant difference at *P* < 0.001 between the fishes that are exposed to arsenic only compared to those that were maintained in tap water with 0.1% DMSO, those that were treated with crude extract and exposed to arsenic, and those that were treated with the crude extract only. Significant difference at *P* < 0.001 was also observed between the fishes that were maintained in tap water and the fishes that were treated with crude extract only. There is significant difference at *P* > 0.01 between the fishes that were maintained in tap water and the fishes that were treated with crude extract and exposed to arsenic. The result also shows significant difference at *P* < 0.05 between the fishes that were treated with crude extract only and those that were treated with crude extract followed by exposure to arsenic (Figure 6).

#### 3.2.7. Result of Butyrylcholinesterase Inhibition

**Figure 7:**
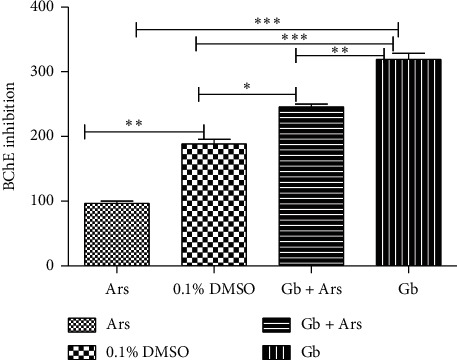
Butyrylcholinesterase inhibition assay of adult Javanese medaka treated with arsenic, crude extract, and arsenic and crude extract. The percentage of butyrylcholinesterase inhibition is shown versus concentration of the tested sample (arsenic, crude extract, and crude extract and arsenic). ^*∗*^*P* < 0.001, *P* < 0.01, and *P* < 0.05 represents significant different values of the control group and the group exposed to arsenic only, crude extract and arsenic, and crude extract only. The values represent mean ± SD from three (*n* = 3) independent experiments.

Result of cholinesterase inhibition shows a significant difference at *P* < 0.01 between the fishes that were exposed to arsenic only compared to those that were maintained in tap water with 0.1% DMSO. Significant difference at *P* < 0.001 was also observed between the fishes that were exposed to arsenic only and the fishes that were treated with crude extract followed by exposure to arsenic, as well as the fishes that were treated with crude extract only. There is significant difference at *P* < 0.001 between the fishes that were maintained in tap water and the fishes that were treated with crude extract only. The result also shows significant difference at *P* < 0.01 between the fishes that were treated with crude extract only and those that were treated with crude extract followed by exposure to arsenic. Significant difference at *P* < 0.05 was also recorded between the fishes that were maintained in tap water and the fishes that were treated with crude extract followed by exposure to arsenic (Figure 7).

#### 3.2.8. Result of Propionylcholinesterase Inhibition

**Figure 8:**
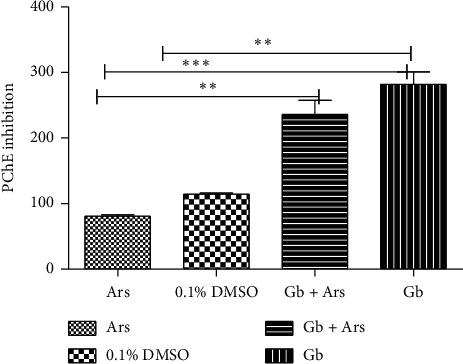
Propionylcholinesterase inhibition assay of adult Javanese medaka (*Oryzias javanicus*) treated with arsenic, crude extract, and arsenic and crude extract. The percentage of propionylcholinesterase inhibition is shown versus concentration of the tested sample (arsenic, crude extract, and crude extract and arsenic). ^*∗*^*P* < 0.001, *P* < 0.01, and *P* < 0.05 represents significant different values of the control group and group exposed to arsenic only, crude extract and arsenic, and crude extract only. The values represent mean ± SD from three (*n* = 3) independent experiments.

Result of propionylcholinesterase inhibition shows a significant difference at *P* < 0.001 between the fishes that were exposed to arsenic only and those were treated with crude extract only. Significant difference at *P* < 0.01 was also observed between the fishes that were exposed to arsenic only and the fishes that were maintained in tap water, as well as the fishes that were treated with crude extract followed by exposure to arsenic. There is significant difference at *P* < 0.01 between the fishes that were maintained in tap water and the fishes that were treated with crude extract only (Figure 8).

#### 3.2.9. Result of High Performance Liquid Chromatography

**Figure 9:**
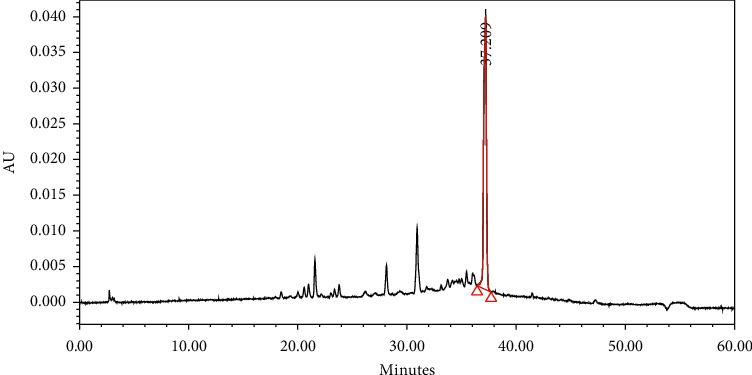
High performance liquid chromatography profile of the crude extract showing vitexin identified at 21.834 minutes (retention time).

Identification of bioactive compounds in the crude extract using HPLC method shows the presence of vitaxin at 21.834 minutes (retention time). The result also shows the presence of various compounds that were unable to be identified due to the unavailability of standard solution (Figure 9).

## 4. Discussion

Traditional medicines are naturally derived from plant bioactive substances with minimal or no industrial processing. They have been used to cure or prevent illness within local or regional healing practices [24]. This work was design to evaluate toxicity and anticholinesterase effect of *Ginkgo biloba* 80% methanolic seed extract.

The yield obtained at the end of the extraction is quite sufficient to conduct the experiment as designed. There are differences in percentage yield among different solvents used for extraction. A previous study recorded different solvents with significant effects on the extractable solids yield of *S. chinensis* root. The study revealed the highest extractable solids (15.6%) with absolute methanol followed by 50% ethanol, 50% methanol, and 50% acetone (14.3%, 12.3%, and 12.2%, resp.). Aqua solvent extracted half of extractable solids in comparison with absolute methanol, whereas 100% ethanol and 100% acetone only extracted 25% of extractable solids extracted by 100% methanol [25]. The result showed that recovery yields of crude semisolid extract prepared from *S. chinensis* root was significantly affected by the extraction solvents. The findings were in agreement with previous research on *Limnophila aromatica* [26] and *Phoenix dactylifera* L. [27] whereby the variation in percentage yield can be explained by the difference in solubility of different compounds in the sample.

Increase of arsenic concentration is responsible for the increase of mortality rate as shown in the subacute and subchronic toxicity results (Figures 1 and 2). The studies showed that chronic exposure to arsenic through well waters has led to peripheral vascular disease referred to as black foot disease [28]. Chronic exposure to inorganic arsenic may lead to various cardiovascular disorders such as atherosclerosis, hypertension, ischemic heart diseases, and ventricular arrhythmias [29]. The ability of arsenic to stimulates nicotinamide adenine dinucleotide phosphate (NADPH) oxidase present in the plasma membrane of vascular endothelial cells and vascular smooth muscle cells (VSMC) causing increase in the generation of reactive oxygen species (ROS) such as superoxides and hydrogen peroxide has also been reported [30]. ROS generation due to exposure to arsenic and nitric oxide (NO) made up peroxynitrite which is a strong oxidant that mediates inflammatory process such as cyclooxygenase-2 [31].

Survival rate of the exposed fishes decreases with increasing concentration of crude extract in both acute and chronic toxicity test (Figures 3 and 4). From this study, it is clearly shown that most of the mortalities were caused by the effect of bioactive constituent present in the crude extract in either high concentrations as in acute toxicity test or cumulative effect as in chronic toxicity test. Although most of the bioactive constituents (polyphenol) present in the plant crude extract mainly have medicinal effect, some researchers also revealed the effect of phenolic compounds such as alkaloid to be detrimental to body cells, tissues, and organs especially if taken in high concentration or used over a long period of time [32]. Previous studies showed that most of the anti-inflammatory agents (flavonoid) cause gastroduodenal mucosal injury resulting from deleterious effect of gastric acid overwhelming the normal defensive properties of the mucosa when used in high concentration [33]. The possibility of systemic effects resulting from the inhibition of endogenous prostaglandin synthesis was also reported. Inhibition of prostaglandin may affect the availability of the epithelial mucus, secretion of bicarbonate, mucosal blood flow, epithelial proliferation, and mucosal resistance to injury [34]. Several works of literature on *in vivo* toxicity study reported the effect of extract with anti-inflammatory effect to affect vital organs such as liver, kidney, heart, spleen, and brain [35]. Damage to these vital organs due to the high concentration of phenolic compounds or their cumulative effect over time may be the main causes of the death in this experimental study.

Cholinesterase refers to the family of enzymes that speed up the hydrolysis of the acetylcholine (ACh) neurotransmitter into choline and acetic acid, a reaction necessary to allow a cholinergic neuron to return to its resting state after activation [36]. Anticholinesterases or AChE inhibitors inhibit the enzyme cholinesterase from hydrolyzing ACh, increasing both the level and duration of the neurotransmitter action [37]. Decrease in cholinesterase activities was observed in fishes that were exposed to arsenic only. There is slight difference in cholinesterase inhibition between fishes that were treated with crude extract only and the fishes that were treated with crude extract followed by exposure to arsenic (Figures 6–8). Slight decrease in cholinesterase activity in fishes that were treated with crude extract followed by exposure to arsenic may be due to the effect of arsenic. Several studies reported the effect of arsenic in inhibiting cholinesterase activities. Chandravanshi et al. in an experimental study, reported the ability of arsenic to cause significant decrease in serum acetylcholinesterase activities in in a dose-dependent manner [38]. This result is also in agreement with the previous studies that demonstrated a decreased activity of acetylcholinesterase in neuroblastoma cells of mice [39], in arsenic induced rat whole brain [40], and in two models of fish [41]. Increase of cholinesterase activities in fishes treated with crude extract followed by administration of arsenic may be due to the neutralizing effect of the phenolic compounds present in the crude extract. Several bioactive compounds were reported to reverse the effect of cholinesterase reduced activities. Alkaloid is among the phenolic compounds that act by selective, competitive, and rapidly reversible AChE inhibition that interacts with the anionic subsite, as well as with the aromatic gorge [42]. The compound is also an allosteric ligand at nicotinic cholinergic receptors inducing their modulation. Alkaloid interacts with the nicotinic receptor at binding sites separate from those for ACh and nicotinic agonists and acts specifically to enhance the activity (sensitize) of nicotinic receptors in the presence of ACh [43].

Vitexin was the identified crude extract using HPLC method. Although there is no online literature that reported the identification or isolation of vitexin in any part of *Ginkgo biloba*, several studies revealed the presence of various phenolic compounds in this plant. Hence this result is not in line with the work reported by [44] who identified several flavonoids in *Ginkgo biloba* extract such as ginkgolide (A, B, C, J), quercetin, and isorhamnetin but not vitexin. It has been reported that vitexin (50 *μ*M) was able to prevent the formation of ROS and NRS which are the markers of protein and lipid oxidation, carbonyl protein, and malondialdehyde [45]. It also reduced loss of membrane potential by a mechanism of increased mitochondrial biogenesis, such as induction of the expression of antioxidant genes nuclei factor 2 (Nrf-2) and heme oxygenase-1 (HO-1) with increased GSH level [46].

## 5. Conclusion

Plant crude extract showed low toxicity effects on Javanese medaka and high cholinesterase inhibition activities. These properties contribute more to the medicinal use of this plant or its benefit to be used as natural medicine/supplements. The crude extract may contain high percentage of other phenolic compounds beside vitexin.

### 5.1. Recommendation

Toxicity screening of this extract on mammals such as mice and rats to reaffirm their toxicity profile and antioxidant screening using DPPH and FRAP assays is recommended. Phytochemical studies and isolation of bioactive compound such as vitexin and isovitexin from crude extracts are strongly recommended.

## Data Availability

The data are available from Ibrahim Maina Hassan but with the permission of the corresponding author, Dr. Syahida Ahmad.
